# Supplementary material for: The effects of genital myiasis on the diversity of the vaginal microbiota in female Bactrian camels
Source: BMC Vet Res. 2022 Mar 5;18:87. doi: 10.1186/s12917-022-03189-5 (PMC8897907; doi:10.1186/s12917-022-03189-5)
Supplement: Supplementary file 5 — Additional file 5. [file 12917_2022_3189_MOESM5_ESM.zip › MPL201709200_16s_yy/Treat1/D01_rda/biplot.pdf]

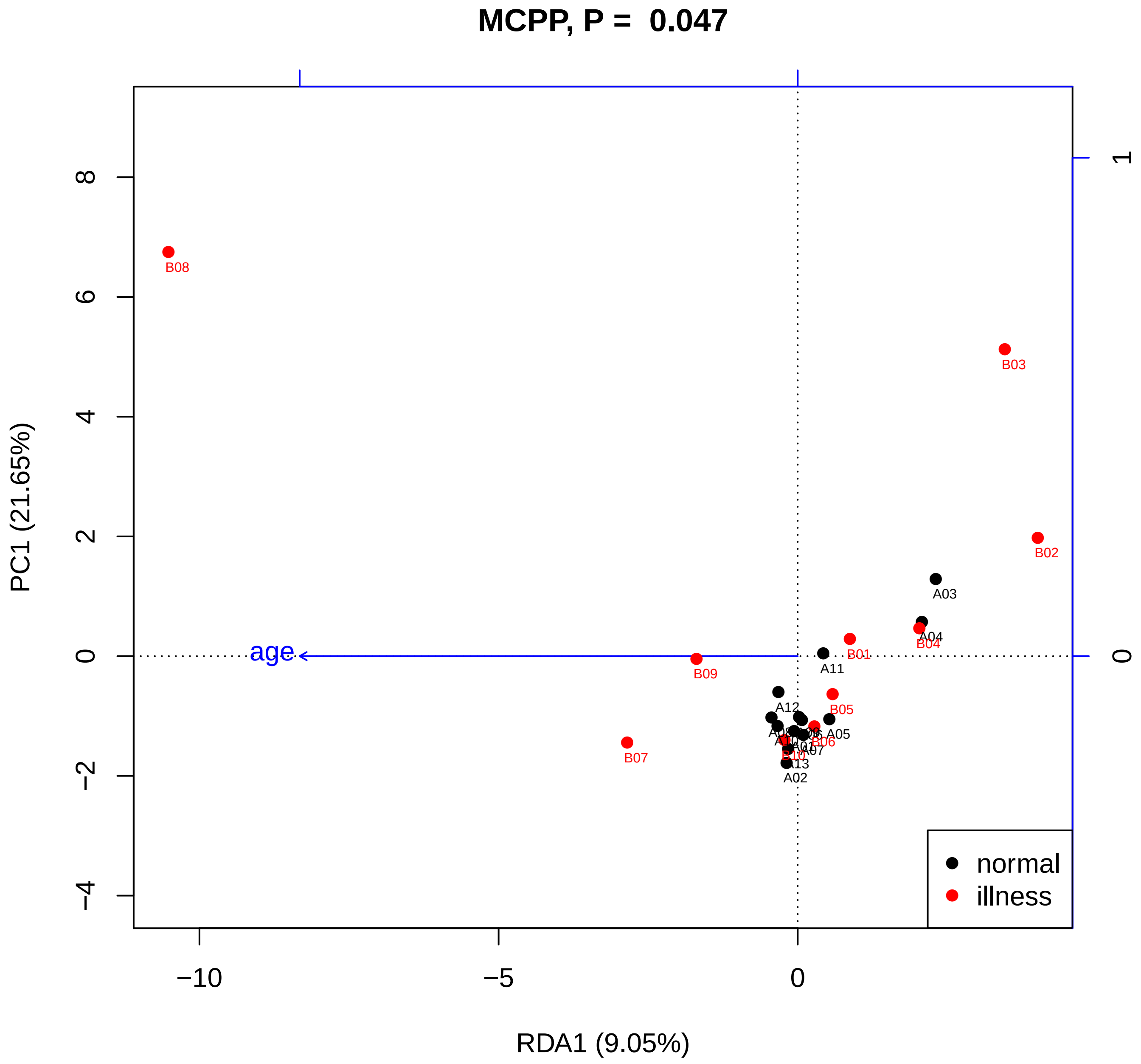
MCPP, P = 0.047
RDA1 (9.05%)
PC1 (21.65%)
age
normal
illness
B08
B03
B02
A03
A04
B04
B01
A11
B09
A12
B05
A05
B06
B07
B10
A13
A02
A07
−10
−5
0
−4
−2
0
2
4
6
8
1
